# Further characterization of adrenocortical and thyroid hormone concentrations of leatherback turtles (*Dermochelys coriacea*) under various stressors, including validation of a plasma aldosterone assay

**DOI:** 10.1093/conphys/coae083

**Published:** 2024-12-14

**Authors:** Charles J Innis, Katherine M Graham, Justin R Perrault, Craig A Harms, Emily F Christiansen, Kara L Dodge, Elizabeth A Burgess

**Affiliations:** Anderson Cabot Center for Ocean Life, New England Aquarium, 1 Central Wharf, Boston, MA 02110, USA; Anderson Cabot Center for Ocean Life, New England Aquarium, 1 Central Wharf, Boston, MA 02110, USA; Loggerhead Marinelife Center, 14200 US Highway 1, Juno Beach, FL 33408, USA; Department of Clinical Sciences, College of Veterinary Medicine, North Carolina State University, 1060 William Moore Drive, Raleigh, NC 27606, USA; Center for Marine Sciences and Technology, North Carolina State University, 303 College Circle, Morehead City, NC 28557, USA; Department of Clinical Sciences, College of Veterinary Medicine, North Carolina State University, 1060 William Moore Drive, Raleigh, NC 27606, USA; Center for Marine Sciences and Technology, North Carolina State University, 303 College Circle, Morehead City, NC 28557, USA; North Carolina Aquariums, 3125 Poplarwood Court, Raleigh, NC 27604, USA; Anderson Cabot Center for Ocean Life, New England Aquarium, 1 Central Wharf, Boston, MA 02110, USA; Anderson Cabot Center for Ocean Life, New England Aquarium, 1 Central Wharf, Boston, MA 02110, USA

**Keywords:** Capture, corticosterone, endocrine, entanglement, nesting, stranding, thyroxine

## Abstract

Leatherback turtles (*Dermochelys coriacea*) are endangered by anthropogenic threats. Characterizing the physiologic response of leatherback turtles under various stressors may inform conservation strategies. In this study, a commercially available enzyme immunoassay for aldosterone was validated for leatherback turtle plasma, and it was used with previously validated assays for corticosterone and free thyroxine (fT4) to evaluate the physiologic status of leatherback turtles that were entangled in fishing gear, stranded on shore, nesting or intentionally captured at sea during ecologic studies. Mean aldosterone concentrations were significantly higher in entangled turtles (156 ± 102 pg/ml), stranded turtles (274 ± 165 pg/ml) and intentionally captured turtles (457 ± 464 pg/ml) than in nesting females (23 ± 16 pg/ml). In contrast, nesting females had higher fT4 (2.9 ± 0.6 pg/ml) compared to entangled turtles (0.8 ± 0.9 pg/ml), stranded turtles (0.7 ± 0.8 pg/ml) and intentionally captured turtles (0.3 ± 0.2 pg/ml). Corticosterone concentrations were significantly higher in stranded individuals (10.9 ± 6.6 ng/ml) compared with nesting (3.8 ± 2.0 ng/ml) and intentionally captured turtles (3.6 ± 2.5 ng/ml), with intermediate levels in entangled turtles (5.1 ± 2.8 ng/ml). This study provides additional insight into the variable physiologic status of leatherback turtles under the influence of different anthropogenic and natural stressors, and it provides an additional tool to evaluate the role of aldosterone in the acute stress response and health of endangered sea turtle species.

## Introduction

The leatherback turtle (*Dermochelys coriacea*) is the last surviving species of the *Dermochelyidae*, a family originating in the Cretaceous period ~100 million years ago (Hirayama and Chitoku, 1996). It is the largest and most highly migratory species amongst extant turtles. Leatherback turtles are listed globally as Vulnerable (high risk of extinction) by the International Union for the Conservation of Nature, with four of its seven regional management units categorized as Critically Endangered (extremely high risk), one as Endangered (very high risk) and two as Data Deficient (IUCN, 2023). Major risks to leatherback turtle populations include injury, illness and death secondary to fisheries interactions, habitat loss, watercraft trauma, plastic ingestion and other anthropogenic activities (Mrosovsky *et al.,* 2009; Stewart *et al.,* 2016; Hamelin *et al.,* 2017; Foley *et al.,* 2019; World Wildlife Fund, 2019; Dodge *et al.,* 2022; Chevallier *et al.,* 2023).

Veterinarians and sea turtle biologists have gathered substantial physiologic data for healthy leatherback turtles at foraging areas and nesting beaches, including hematologic, clinical biochemical and endocrine data (Rostal *et al.,* 2001; Deem *et al.,* 2006; Harms *et al.,* 2007; Innis *et al.,* 2010, 2014; Harris *et al.,* 2011; Honarvar *et al.,* 2011; Perrault *et al.,* 2012, 2014; Hunt *et al.,* 2016; Stacy *et al*., 2019; Stewart *et al.,* 2012, 2023). The physiologic status of leatherback turtles that were entangled in fishing gear or stranded on shore has been evaluated less thoroughly, but available data indicate altered metabolic states [e.g. elevated plasma betahydroxybutyrate and free thyroxine (fT4); reduced blood urea nitrogen (BUN) and triglycerides], adrenocortical stress response (elevated corticosterone) and other hematologic and plasma biochemical changes (Innis *et al.,* 2010; Hunt *et al.,* 2016).

The hypothalamic–pituitary–adrenal axis (HPAA) of vertebrates involves a cascade of hormones sequentially released by the hypothalamus (corticotropin-releasing hormone, CRH), pituitary (adrenocorticotropic hormone, ACTH) and adrenal glands (glucocorticoids and mineralocorticoids). Adrenal glucocorticoid hormones such as corticosterone affect important physiologic functions, especially in response to stressors. Glucocorticoids alter behaviour and facilitate energy availability for critical tissues, which may be adaptive during response to stressors, whilst inhibiting growth, reproduction and immune response, which may be of lesser short-term importance (reviewed by Romero and Butler, 2007). Adrenal mineralocorticoids, including aldosterone play critical roles in feedback loops, such as the renin-angiotensin-aldosterone system, that regulate plasma electrolyte concentrations, blood volume, and blood pressure (reviewed by Fournier *et al.,* 2012). Thyroid hormones, such as thyroxine, affect many processes in vertebrates, including metabolic rate, energy mobilization during exercise, regulation of body temperature and seasonal transitions (reviewed by Zwahlen *et al.,* 2024). There has been limited study of glucocorticoid and thyroid activity for leatherback turtles, and adrenal mineralocorticoid activity has not yet been studied for this species (Rostal *et al.,* 2001; Deem *et al.,* 2006; Perrault *et al.,* 2012; Hunt *et al.,* 2016). As in other vertebrates, aldosterone is the major mineralocorticoid in reptiles (Macchi and Phillips, 1966; Mehdi and Carballeira, 1972; Duggan and Lofts, 1978; Brewer and Ensor, 1980; Uva *et al.,* 1982; Balment and Loveridge, 1989). However, there are very limited data for plasma aldosterone concentrations of sea turtles (Ortiz *et al.,* 2000; Innis *et al.,* 2024). Studies of several lizard species indicate that adrenocortical cells *in vitro* have labile responsiveness to ACTH based on species, season, sex, reproductive status and food intake, with aldosterone response being proportionately greater than corticosterone response in some circumstances (Carsia *et al.,* 2023). Thus, it has been suggested that both plasma corticosterone and aldosterone be evaluated in studies of the HPAA for free-ranging animal populations (Carsia *et al.,* 2023). For several marine mammal species, plasma aldosterone concentrations correlate positively with glucocorticoid concentrations, increasing under the influence of stressors (Burgess *et al.,* 2017; Champagne *et al.,* 2018; Bergfelt *et al.,* 2020).

In the present study, we validated an immunoassay to determine plasma aldosterone concentrations for leatherback turtles. We then measured aldosterone alongside previously validated assays for corticosterone and fT4 to further characterize the endocrine status of leatherback turtles that were assessed under varied circumstances. We hypothesized that (1) aldosterone could be reliably measured in leatherback turtle plasma, (2) plasma aldosterone concentrations would be higher in stranded and entangled turtles compared to healthy free-ranging individuals and (3) aldosterone concentrations would co-vary with corticosterone and thyroxine.

## Materials and Methods

### Animals and samples

Authorization for evaluation and management of turtles from which samples were collected included United States Fish and Wildlife Service permits ES69328D, TE697823; National Marine Fisheries Service permits 15 672, 1557, 21 301; New England Aquarium IACUC proposals 06-03, 2013-07, 2019-03, 2016-03; University of New Hampshire IACUC proposal 090402; University of Massachusetts Amherst IACUC proposal 2010-0019; Florida Fish and Wildlife Conservation Commission Marine Turtle Permit 205; and serial North Carolina permits 04ST42 through14ST42. Disentanglement responses were performed under the authorization of Final Rule 50 CFR Part 222.310 issued by the National Oceanic and Atmospheric Administration.

We acquired an archived, frozen (−80°C), heparinized plasma sample from each of 56 live leatherback turtles that were assessed and sampled under one of four circumstances: nesting, entangled in fishing gear, stranded on shore, and presumed healthy individuals that were sampled at sea during ecologic studies (hereafter referred to as ‘intentionally captured’ turtles). The sample set included 30 nesting females, 6 entangled individuals, 10 stranded individuals and 10 intentionally captured individuals. Evaluation and sampling were conducted at Juno and Jupiter Beaches, Florida, USA, for nesting females (2019–20); Massachusetts, USA, for all entangled (2007–21), intentionally captured (2008–12) and four of the stranded individuals (2011–18); and North Carolina, USA, for six of the stranded individuals (2004–14). Curved carapace lengths ranged from 115 to 164 cm (mean ± SD, 149 ± 10 cm; median, 150 cm, Supplementary Table 1). The sex of entangled, stranded and captured turtles was identified by external dimorphism, necropsy (conducted after blood collection and euthanasia of moribund stranded individuals) or was categorized as undetermined; 12 were female, 9 were male and 5 were of undetermined sex (Supplementary Table 1).

Methods used for blood collection and plasma preparation for leatherback turtles sampled in Massachusetts and Florida have been previously described (Innis *et al.,* 2010, 2014; Perrault *et al.,* 2012; Hunt *et al.,* 2016). Heparinized blood samples from North Carolina were collected just prior to euthanasia and held in a cooler with ice until brought to a laboratory for centrifugation within 4 h of collection. When known, the time of blood collection relative to the time of initial handling was recorded for entangled and intentionally captured turtles, although the time of initial entanglement was unknown. Similarly, the time of initial stranding was unknown, thus the interval between stranding and blood collection could not be determined. Nesting females were encountered at variable times in the nesting process (emergence through egg laying) and the time of blood collection was not recorded for every case, thus the interval between emergence and blood collection could not consistently be determined. However, 14 individuals had data available to determine the interval between body pit initiation (the first phase of nest construction) and blood collection, which provided a proxy for emergence to blood collection interval (Supplementary Table 1).

### Hormone analysis

Aldosterone concentrations were measured using a commercially available enzyme immunoassay (#K052; Arbor Assays, Ann Arbor MI) following methods of a previous sea turtle study (Innis *et al.,* 2024). Prior to hormone measurement, plasma samples were extracted according to the manufacturer’s guidelines. In summary, 250 μl of plasma was extracted by combining with an equal volume of 250 μl ethyl acetate in a glass tube. The mixture was vortexed for 30 s and then allowed to separate into layers. The top organic layer containing the hormones was removed and transferred to a clean collection tube. These extraction steps were repeated twice more, combining supernatant solutions. The resulting sample extract was dried under air and reconstituted in 250 μl of assay buffer (#X065, Arbor Assays) for hormone analysis. Analytical validations for aldosterone included testing: (1) parallelism between serially diluted plasma extract pool (dilution range tested: neat, 1:1–1:16) and assay standards; and (2) accuracy of assay standard concentrations spiked with pooled plasma extract compared to unspiked standards (i.e. matrix effect). For aldosterone measurement, extracted plasma samples were assayed undiluted (neat, 1:1) following the manufacturer’s overnight protocol. To improve assay measurement at lower concentrations, the standard curve was diluted an additional step, resulting in seven standards (range: 0.98–4000 pg/ml). Assay plates were read at optical density 450 nm using a microplate reader with Epoch Gen5™ software (BioTek Instruments, Inc., Winooski, VT). For measurement of corticosterone and fT4 we used commercial ^125^I radioimmunoassay kits (#07-120 103, #06B-257 214, MP Biomedicals, Solon, OH, USA) that were previously validated for use with leatherback turtle plasma (Hunt *et al.,* 2016). Samples were assayed unextracted at 1:10 dilution in steroid diluent for corticosterone assay, and neat (undiluted, 1:1) for fT4 assay. Immunoassays were performed with minor modifications to manufacturer’s protocol (see Hunt *et al.,* 2016). Specifically, the corticosterone assay was conducted at 50% volume and an additional low standard was included in both assays (i.e. seven corticosterone standards, range: 0.0625–5 ng/ml; six fT4 standards, range: 1.5–120 pg/ml). Samples were quantified using a Wallac 1470 Wizard® gamma counter (PerkinElmer Life Sciences, Waltham, MA, USA) on a 2-min count time. Concentrations of corticosterone and fT4 in plasma samples from some of the leatherback turtles reported in this study have been presented elsewhere (*n* = 19 samples for corticosterone and *n* = 21 samples for fT4) (Hunt *et al.,* 2016) and are used here to further interpret aldosterone results.

Assay standards, controls and samples were all measured in duplicate. Coefficient of variation between duplicate sample measurements was kept <10%. Assay precision and reproducibility were monitored by measuring high (~30% binding)- and low (~70% binding)-concentration control samples in each assay. Inter-assay coefficients of variation for high and low controls were: 7 and 4% for aldosterone (*n* = 4 assays); 5 and 5% for corticosterone (*n* = 6 assays); and 3 and 8% for fT4 (*n* = 4 assays). Antibody cross-reactivity can be referenced in the manufacturers’ protocols for all assays. Final corticosterone results are reported as nanograms of immunoreactive hormone per millilitre of plasma (ng/ml); whereas final fT4 and aldosterone data are reported as picograms of immunoreactive hormone per millilitre of plasma (pg/ml).

### Statistical analysis

Parallelism results were plotted as percentage of antibody bound against relative dose (log-scale), with the neat dilution (1:1) assigned a nominal value and each subsequent dilution assigned a relative dose of half the previous dilution. The difference of slope between the linear portion of sample binding curve compared to the standard curve was assessed with an *F* test (Fleetwood *et al.,* 2015). Accuracy results were plotted as observed dose versus known standard dose and assessed using linear regression, with acceptable accuracy defined as *R^2^* > 0.95 and slope = 1.0 (0.8–1.2 acceptable range).

Hormone values for fT4 were sometimes below the limit of assay detection (*n* = 16); such samples were assigned a nominal value that was half of the quantification limit (i.e. fT4 value of 0.23 pg/ml) to avoid non-random missing data in statistical analyses (Beal, 2001). Aldosterone and corticosterone concentrations were transformed using the common logarithm, log_10_, to adjust for a skewed, non-normal distribution. Levene tests were conducted for all variables to check for homogeneity of variance, using Welch’s correction for unequal variances. To examine circumstantial effects on the physiologic state of the turtles, we compared hormone concentrations amongst groups (nesting, entangled, stranded and intentionally captured) using two-way analysis of variance (ANOVA), which included sex and an interaction term (sex × group); a *post hoc* Tukey test was used to locate differences. We performed Spearman’s rank–order correlations to evaluate the relationship between each hormone analyte for each circumstance. Differences were considered significant at α < 0.05. Statistical analyses were performed using the statistical programme IBM SPSS Statistics for Mac OS, version 28 (IBM Corp, Armonk, NY, USA).

## Results

**Figure 1:**
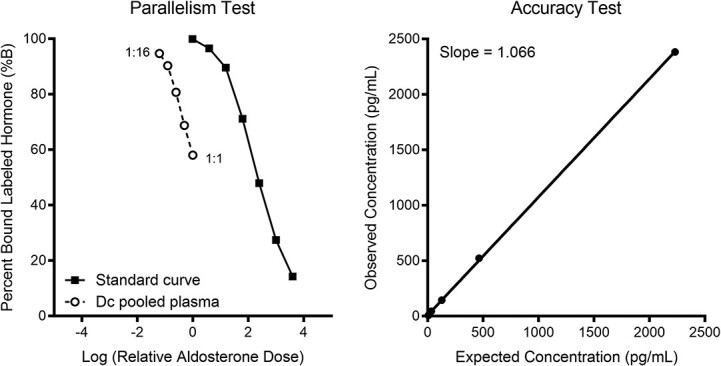
Validation plots for measuring aldosterone in plasma extracts from leatherback turtles using enzyme immunoassay. Results demonstrate (left) close parallelism between serially diluted samples (1:1–1:16) to the aldosterone standard reference curve (3.9–4000 pg/ml; black squares); and (right) good accuracy demonstrated by the positive linear relationship of known aldosterone concentration (expected) against apparent concentration in spiked samples, with a slope of 1.0.

Validation of the aldosterone assay for leatherback turtle plasma was successful (Fig. 1). The assay was able to accurately measure a range of aldosterone concentrations (*R*^2^ = 0.99, slope = 1.07), and there was no interfering plasma matrix effect (i.e. strong parallelism, *F*_1,9_ = 0.06, *p* = 0.81).

**Figure 2:**
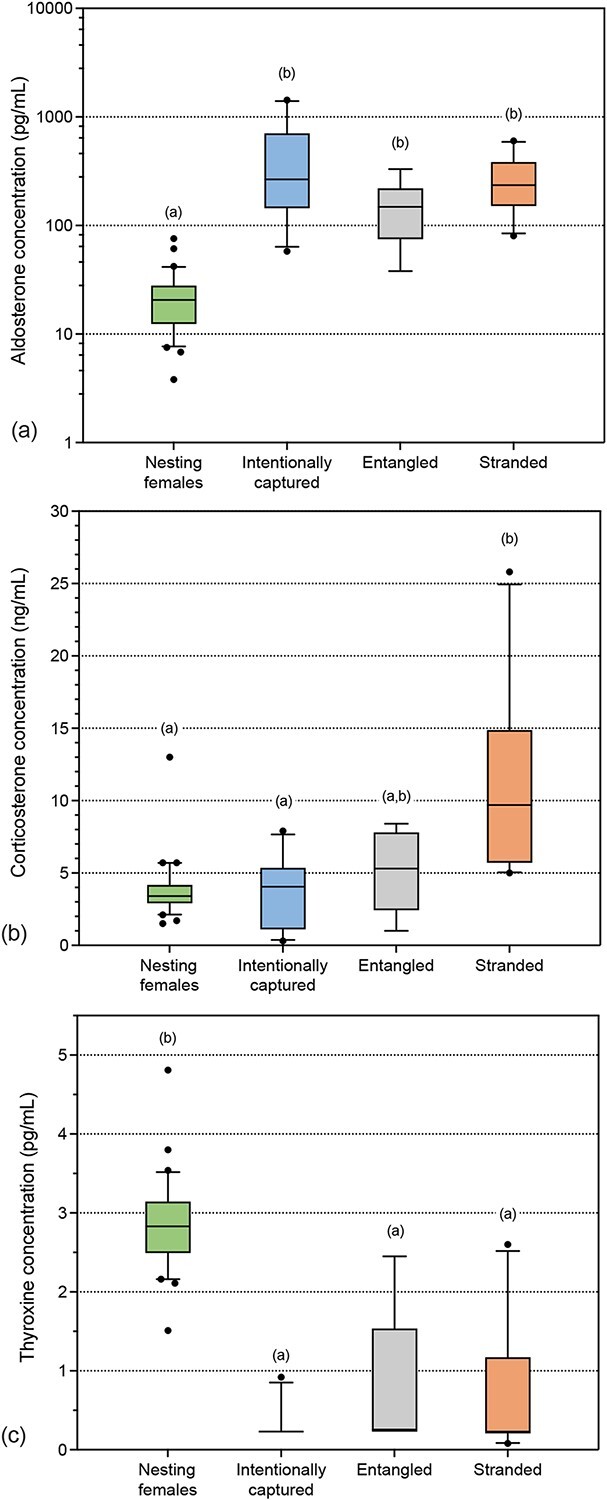
Differences in plasma (a) aldosterone (log-scale, pg/ml), (b) corticosterone (ng/ml) and (c) fT4 (pg/ml) hormone concentrations for leatherback turtles that were nesting (*n* = 30), intentionally captured (*n* = 10), entangled in fishing gear (*n* = 6) or stranded on shore (*n* = 10). For boxplots, the line inside the box indicates the median value, the height of the box encompasses the distance between the 25th and 75th percentiles and the whiskers delineate extreme observations. Outliers are marked with a circle (>1.5 × interquartile range). Each hormone showed significant variation with circumstances as assessed using ANOVA. Different letters denote significantly different groups at *P* < 0.05 as determined by Tukey’s *post hoc* tests.

Hormone data for individual turtles are provided in Supplementary Table 1. All hormones measured were significantly influenced by circumstance (Fig. 2, ANOVA, aldosterone: *F_3,51_* = 35.52, *P* < 0.001; corticosterone: *F_3,51_* = 9.96, *P* < 0.001; and fT4: *F_3,51_* = 25.76, *P* < 0.001). Nesting females had significantly lower aldosterone and higher fT4 concentrations compared to all other groups (*post hoc* comparison, all *P* < 0.001). Mean (± SD) aldosterone values were at least 6-fold lower in nesting females (23 ± 16 pg/ml) than entangled turtles (156 ± 102 pg/ml), stranded turtles (274 ± 165 pg/ml) and intentionally captured turtles (457 ± 464 pg/ml). In contrast, nesting females had higher fT4 values (2.9 ± 0.6 pg/ml) compared to entangled turtles (0.8 ± 0.9 pg/ml), stranded turtles (0.7 ± 0.8 pg/ml) and intentionally captured turtles (0.3 ± 0.2 pg/ml). For corticosterone, concentrations were highest in stranded individuals (10.9 ± 6.6 ng/ml) compared with nesting (3.8 ± 2.0 ng/ml; *post hoc* comparison, *P* < 0.001) and intentionally captured turtles (3.6 ± 2.5 ng/ml; *post hoc* comparison, *P* < 0.001), with intermediate levels measured in entangled turtles (5.1 ± 2.8 ng/ml; *post hoc* comparison, *P* > 0.05). There were no significant differences between sexes (ANOVA, *P >* 0.05) or interaction effect amongst groups for all hormones measured (ANOVA, *P* > 0.05). Aldosterone concentrations were positively correlated with corticosterone concentrations for intentionally captured turtles (*r_s_* = 0.842, df = 8, *P* = 0.002). There were no correlations amongst aldosterone, corticosterone nor fT4 for any other circumstances.

## Discussion

This study provides the first data for plasma aldosterone concentrations of leatherback turtles. The aldosterone assay used in this study can easily be used for future clinical and research applications because it is commercially available and does not involve radioisotopes. The assay has now been validated for two sea turtle species (Innis *et al.,* 2024), suggesting that it has potential for validation in other reptile species.

Plasma aldosterone concentrations of vertebrates can vary widely. For example, an order of magnitude variation is seen within aldosterone reference intervals for both humans and dogs, with concentrations generally ranging from the tens to low hundreds pg/ml (Meunier *et al.,* 2015; Eisenhofer *et al.,* 2017; Tan *et al.,* 2020; Kai *et al.,* 2022). Aldosterone concentrations in stranded, entangled and intentionally captured leatherback turtles in this study were often higher than maximal values within these mammalian reference intervals, whilst concentrations in nesting females were more typical of resting concentrations in mammals. Amongst reptiles, limited data for plasma aldosterone concentrations show great variability, possibly due to differences in acclimation to laboratory conditions, animal and sample handling procedures, sampling methods, etc. In general, however, plasma aldosterone concentrations amongst nesting leatherback turtles were within the same order of magnitude of those documented for captive Hermann’s tortoises (*Testudo hermanni*; mean 82 pg/ml, *n* = 6) and captive Nile crocodiles (*Crocodylus niloticus*; mean 75 pg/ml, *n* = 4); whilst concentrations for stranded, entangled and intentionally captured leatherback turtles were an order of magnitude greater, consistent with data for captive North African spiny tailed lizards (*Uromastyx acanthinura*; mean 360 pg/ml, *n* = 18), captive shingleback skinks (*Tiliqua rugosa*; mean 317 pg/ml, *n* = 24) and captive sand monitors (*Varanus gouldii*; mean 459 pg/ml (Bradshaw and Grenot, 1976; Bradshaw and Rice, 1981; Uva *et al.,* 1982; Balment and Loveridge, 1989). Amongst sea turtles, aldosterone concentrations have previously only been reported for Kemp’s ridley turtles in laboratory and hospital settings (Ortiz *et al.,* 2000; Innis *et al.,* 2024). Plasma aldosterone concentrations in nesting leatherback turtles were similar or moderately higher in comparison to clinically healthy Kemp’s ridley turtles (eating, active, normalized plasma biochemistry) that had been rehabilitated for 1–2 months after exposure to the Deepwater Horizon oil spill (Innis *et al.,* 2024; mean 8.7 pg/ml, *n* = 30). The substantially greater plasma aldosterone concentrations of stranded, entangled and intentionally captured leatherback turtles were similar to those documented in Kemp’s ridley turtles upon initial hospitalization after the Deepwater Horizon oil spill (Innis *et al.,* 2024; mean 549 pg/ml, *n* = 30) and in Kemp’s ridley turtles held under laboratory conditions (Ortiz *et al.,* 2000; mean 351 pg/ml, *n* = 4).

Aside from the present study, we are aware of only one other study that evaluated plasma aldosterone concentrations in free-ranging reptiles. Faulkner *et al.* (2021) documented plasma aldosterone concentrations for juvenile American alligators in Louisiana during seasonally variable environmental salinity and found that for females, but not males, aldosterone concentrations were negatively correlated with salinity, with monthly mean concentrations ranging over an order of magnitude between 20 and 460 pg/ml. All other studies of reptile plasma aldosterone concentrations, to our knowledge, have involved animals in laboratory or hospital settings, including studies that demonstrated decreased concentrations in response to sodium loading (Bradshaw and Grenot, 1976; Bradshaw and Rice, 1981; Uva *et al.,* 1982), no change in concentrations in response to salt loading (despite documented hypernatremia, Balment and Loveridge, 1989; Faulkner *et al.,* 2018), increased concentrations in response to water loading (Bradshaw and Grenot, 1976; Uva *et al.,* 1982) or no change in concentrations in a low-salinity environment (Ortiz *et al.,* 2000). Additional experimental studies documented increased plasma aldosterone concentrations in response to exogenous ACTH and decreased concentrations in response to exogenous corticosterone (Vallarino *et al.,* 1985; Morici *et al.,* 1997). In light of these experimental data, the *in vitro* studies of Carsia *et al.* (2023) and the present study, we expect that additional studies of plasma aldosterone in free-ranging reptiles will demonstrate circumstantially variable results influenced by endogenous and exogenous factors such as sex, season, environmental conditions, health status and reproductive status.

Corticosterone concentrations in this study were highest in stranded individuals and were very similar to those previously reported for ‘distressed’ leatherback turtles, including some of the same data that were incorporated in the present study (Hunt *et al.,* 2016; mean 10.1 ng/ml, *n* = 15). Corticosterone concentrations for entangled, intentionally captured and nesting leatherback turtles in this study were similar to previously reported values for healthy individuals of this species, including nesting females [Rostal *et al.,* 2001 (mean ~2.0 ng/ml, *n* = 184), Deem *et al.,* 2006 (maximum 4.0 ng/ml, *n* = 12), Hunt *et al.,* 2016 (mean 5.0 ng/ml, *n* = 17)]. Combined with these previous data, results of the present study suggest that baseline corticosterone concentrations for leatherback turtles are often ≤6 ng/ml, whilst severe stressors may elevate corticosterone into the range of ≥10 ng/ml. Cautious interpretation is needed, however, given the substantial overlap of ranges amongst groups (Fig. 2).

fT4 concentrations in this study were greatest for nesting females, whilst there was no difference in fT4 amongst other circumstances, suggesting that thyroxine may be upregulated for egg production or energy mobilization during the process of nesting. No other fT4 data for nesting females of this species are available, with the only previous data being total thyroxine concentrations (including protein-bound thyroxine) derived from an assay that had not been specifically validated for this species (total thyroxine 0–2.2 μg/dl; Perrault *et al.,* 2012). fT4 was measurable in samples from every nesting female, but concentrations were below the detection limit of the assay in samples from many individuals in other circumstances, especially for intentionally captured turtles (Supplementary Table 1). Hunt *et al.* (2016) demonstrated a significant difference in fT4 concentrations between intentionally captured leatherback turtles (lower fT4, mean 0.05 pg/ml, *n* = 17) in comparison to ‘distressed’ leatherback turtles (entangled, stranded, higher fT4, mean 0.9 pg/ml, *n* = 15) and proposed this as evidence of increased energy mobilization under the effects of a physical stressor. The present study did not detect such differences, possibly due to the large number of samples with undetectable fT4 concentrations. Given the number of results below the assay detection limit, a more sensitive assay may be needed for determination of baseline fT4 concentrations for intentionally captured healthy leatherback turtles. The commercial fT4 assay that has been validated for leatherback turtle plasma (Hunt *et al.,* 2016) is very sensitive in comparison to several other commercially available assays, thus it is likely that greater sensitivity would require development of a customized assay. Future studies might also consider validation and measurement of other forms of thyroid hormone, such as triiodothyronine (T3). We did not evaluate T3 because a previous study found it to be undetectable in plasma of Kemp’s ridley sea turtles, and the fT4 assay used in the present study had already been validated for leatherback turtles.

Previous research on blood gas, blood biochemical, corticosterone and fT4 data indicated that entanglement and stranding in leatherback turtles were more physiologically stressful than intentional capture, as might be intuitively expected (Innis *et al.,* 2010, 2014; Hunt *et al.,* 2016). Nonetheless, capture and handling do appear to induce physiologic changes that could affect the safety and health of the turtle (e.g. hyperkalaemia in some cases, Innis *et al.,* 2014). The present study provides further evidence of the acute effects of stressors, somewhat surprisingly demonstrating significantly higher aldosterone concentrations in intentionally captured leatherback turtles compared to other circumstances. This suggests that aldosterone may play an immediate role in leatherback turtles at the onset of an acute stressor, perhaps even more rapidly than corticosterone, which could be adaptive for the animal during physically challenging situations. Release of aldosterone during an adverse event could support electrolyte and cardiovascular homeostasis. For example, higher aldosterone concentrations may help to maintain normokalaemia when other factors such as acidosis or muscle trauma may promote hyperkalaemia. Cumulatively, results of this study and previous observations for this species allow for general characterization of leatherback turtles’ physiologic status in three major circumstances:

1. Healthy intentionally captured leatherback turtles: These turtles are presumed healthy when captured at sea in seasonal foraging regions. fT4 and corticosterone are relatively low (perhaps baseline). Aldosterone appears to be increased acutely by the capture event and may be accompanied by increases in potassium and lactate (Innis *et al.,* 2014). Elevated potassium concentrations are clinically concerning since hyperkalaemia often carries a poor prognosis for ill sea turtles (Innis *et al.,* 2009; Keller *et al*., 2012; Stacy *et al*., 2013). Whilst shown to be generally safe based on subsequent long-term monitoring of captured animals (e.g. Innis *et al.,* 2010, 2014; Harris *et al.,* 2011; Dodge *et al.,* 2014), the observed physiologic changes support ongoing efforts to maintain efficiency, veterinary oversight and emergency medical gear during leatherback turtle capture and handling procedures.
2. Healthy nesting leatherback turtles: These turtles are presumed healthy when sampled during nesting. Concentrations of corticosterone and aldosterone are relatively low (perhaps baseline). Evidence for altered physiologic status includes relatively high fT4 and glucose and relatively low sodium, chloride and BUN (Deem *et al.,* 2006; Harms *et al.,* 2007; Honarvar *et al.,* 2011; Perrault *et al.,* 2012; Stewart *et al.,* 2012, 2023). These changes may reflect increased energy mobilization, metabolism and muscle activity associated with emergence, nest construction and oviposition (i.e. affecting fT4, glucose), as well as reduced food and seawater ingestion during the breeding and nesting season (i.e. affecting sodium, chloride, BUN) (Perrault *et al.,* 2014). The lack of elevated corticosterone for nesting leatherback turtles was previously described by Rostal *et al.* (2001) as a ‘suppressed’ adrenocortical stress response, perhaps adaptive as a ‘trade-off between survival and reproduction’.
3. Entangled and stranded leatherback turtles: These turtles vary along the spectrum of morbidity, likely related to the duration and severity of the circumstance. Moderately to severely elevated concentrations of aldosterone, corticosterone, fT4, potassium and lactate are common, as well as moderately reduced concentrations of glucose and BUN (Innis *et al.,* 2010). These changes likely reflect the adrenocortical stress response, energy mobilization, muscle exertion, increased metabolism and decreased food intake during these events. Additional evidence for these probable mechanisms includes elevated beta-hydroxybutyrate and reduced triglyceride concentrations in entangled leatherback turtles (Innis *et al.,* 2010).

There are several limitations of our study. Methodologically, like the previous retrospective clinical endocrine study of this species (Hunt *et al.,* 2016), the time of blood sample collection relative to initial disturbance and handling was often unknowable (e.g. unobserved stranding) or prolonged (e.g. intentional capture and disentanglement responses), which likely resulted in altered hormone concentrations relative to true baseline. In other species, glucocorticoid and thyroid hormones can be released within minutes of exposure to a stressor. For example, Romero and Reed (2005) demonstrated that corticosterone may increase within 1.5 min of handling in several avian species, and Gideon *et al.* (2020) demonstrated increased aldosterone in humans within 1 min of an induced psychosocial stressor. fT4 increases over the first 10 min of strenuous exercise in humans (Ciloglu *et al.,* 2005). Beyond basic measurement of hormone concentrations, recent transcriptomic evidence from an avian stress model (immobilized restraint) suggests rapid activation of genes within the central nervous system that concurrently promote glucocorticoid and thyroid hormone cascades (Kadhim and Kuenzel, 2022). Determining the precise sequence, magnitude and duration of the adrenocortical and thyroid response of leatherback turtles to stressors would require additional studies with rapid serial sampling. However, given the size, habitat and legal status of this species, and their absence in captivity, it is not possible to acquire truly baseline samples nor rapid serial samples. That is, free swimming and entangled individuals must be captured and boarded on a vessel for evaluation, which often takes tens of minutes; and individuals that can be accessed more readily (stranded, nesting) are in specific physiologic states that may not reflect baseline values for free-swimming, healthy individuals. Indeed, sampling of nesting females often is only permitted after they have begun oviposition, at which time their physiologic state has likely been altered by the time-consuming processes of emergence and nest construction (as in this study). Whilst investigators can attempt to acquire blood samples as quickly as possible, truly baseline analyte concentrations will not likely be determined for this species without development of remote analysers that can report data for days to months after deployment.

An additional limitation of the present study is the small sample size and variable circumstances of the entangled, stranded and intentionally captured turtle cohorts. This limitation is due to the rarity of such events for this species and limited plasma that remained to be archived after meeting real-time clinical needs. We utilized all available archived samples from the two research groups that conduct the majority of leatherback turtle stranding and entanglement response in the eastern USA, spanning a period of 15 years. As a result of the timespan, multi-state collaboration and circumstances that were required for this study on a highly migratory species, there are inherent covariates such as seasonality, time of day and geographic location, all of which could influence turtles’ metabolic state and/or body temperature, and thus affect hormone concentrations. For example, all nesting females were sampled at night in Florida in April, whilst all entangled and intentionally captured turtles were sampled during the day in Massachusetts in summer, and stranded turtles were sampled during the day in Massachusetts and North Carolina in summer and autumn. Despite these restrictions, this study provides valuable data for leatherback turtles under different circumstances, which can be built upon in future studies.

A final potential limitation of this study is the extended storage duration of some samples, which could have affected hormone integrity. Whilst many samples used in this study had been frozen for only 1–2 years, others had been frozen for up to 14 years. This is likely an acceptable duration, however, since previous studies demonstrated the stability of thyroid hormones and corticosteroids during long-term freezing and freeze–thaw cycles (Kley and Rick, 1984; Mannisto *et al.,* 2007; Lie and Thorstensen, 2018).

In summary, our study provides an additional validated tool to assess the physiologic status of endangered leatherback turtles and provides insight into the variable physiologic status of leatherback turtles under the influence of different anthropogenic and natural stressors. Such tools are useful for veterinarians and managers in understanding the health of individual turtles and for monitoring population-level deviations from baseline. Our results confirm that aldosterone is present as part of the acute adrenal physiologic stress response in this species. Data from this study can be compared to future nesting leatherback turtles in Florida and elsewhere, monitoring for evidence of elevated aldosterone concentrations that could indicate a cryptic stressor. Similarly, conservation ecology studies that intentionally capture leatherback turtles may use aldosterone to monitor the magnitude of capture stress. Observations of elevated aldosterone concentrations during leatherback turtle capture events provide additional support for cautious planning to optimize efficiency and safety. It is also possible that aldosterone may be an important analyte for monitoring the health and physiologic stress in leatherback turtles in the presence of cumulative anthropogenic stressors, including entanglement, vessel strike and ocean industrial activities.

## Supplementary Material

Web_Material_coae083

## Data Availability

The data underlying this article are available in the article and in its online supplementary material.
