# Supplementary material for: Further characterization of adrenocortical and thyroid hormone concentrations of leatherback turtles (Dermochelys coriacea) under various stressors, including validation of a plasma aldosterone assay
Source: Conserv Physiol. 2024 Dec 14;12(1):coae083. doi: 10.1093/conphys/coae083 (PMC11646118; doi:10.1093/conphys/coae083)
Supplement: Web_Material_coae083 [file web_material_coae083.zip › Supp File 1 CONPHYS-2024-032 Innis et al leatherback turtle aldosterone 9 9 24 .pdf]

State	Turtle ID	Circumstance	Month-year	Sex	CCL cm	ALD pg/mL	CORT ng/mL	fT4 pg/mL	Time min
MA	05-07	Entangled	Aug-07	M	143.2	117.4	6.1	2.45	NA
MA	06-07	Entangled	Aug-07	U	123	87.0	2.9	BLD	NA
MA	11-08	Entangled	Aug-08	M	146.4	179.1	8.4	0.28	39
MA	05-09	Entangled	Sep-09	M	155	330.7	4.5	BLD	49
MA	19-003	Entangled	Jul-19	U	142	38.0	1.0	BLD	7
MA	21-005	Entangled	Oct-21	F	145	182.4	7.6	1.23	NA
MA	01-08	Capture	Jul-08	M	149.5	165.8	3.4	0.92	22
MA	03-08	Capture	Jul-08	F	161.5	570.6	7.9	BLD	40
MA	06-08	Capture	Aug-08	U	133.8	115.8	1.1	BLD	36
MA	07-08	Capture	Aug-08	M	153.3	1430.2	5.5	BLD	30
MA	04-09	Capture	Aug-09	U	127.7	1112.8	5.3	BLD	46
MA	01-12	Capture	Aug-12	U	136.7	57.8	0.3	BLD	25
MA	02-12	Capture	Aug-12	F	148	435.3	4.9	BLD	37
MA	04-12	Capture	Aug-12	M	151.6	153.7	4.7	BLD	42
MA	05-12	Capture	Aug-12	F	156	168.4	1.6	BLD	22
MA	06-12	Capture	Aug-12	M	143.5	362.9	1.1	BLD	25
NC	040127	Stranded	Jan-04	M	144	160.4	5.0	1.78	NA
MA	11-007	Stranded	Nov-11	F	137	354.5	9.7	0.08	NA
MA	12-016	Stranded	Sep-12	M	150	601.2	25.8	0.14	NA
NC	130717	Stranded	Jul-13	F	152	274.2	17.2	2.60	NA
MA	13-002	Stranded	Sep-13	F	163.6	306.3	10.4	BLD	NA
NC	140131	Stranded	Jan-14	F	154	168.2	5.4	BLD	NA
NC	140305	Stranded	Mar-14	F	161	80.2	6.4	BLD	NA
NC	140308	Stranded	Mar-14	F	115	195.6	5.8	0.97	NA
NC	150306	Stranded	Mar-15	F	146	472.3	9.7	BLD	NA
MA	18-061	Stranded	Nov-18	F	116	123.4	14.1	0.48	NA
FL	1136	Nesting	Apr-19	F	146	13.8	3.7	2.83	NA
FL	1137	Nesting	Apr-19	F	154	7.5	2.3	2.97	NA
FL	585	Nesting	Apr-19	F	164.2	61.0	3.0	1.51	NA
FL	546	Nesting	Apr-19	F	147.2	11.3	3.6	2.53	NA
FL	1139	Nesting	Apr-19	F	146.4	17.1	3.7	3.30	NA
FL	10	Nesting	Apr-19	F	161.3	13.7	5.7	2.83	NA
FL	622	Nesting	Apr-19	F	150.3	9.1	2.9	2.52	NA
FL	1142	Nesting	Apr-19	F	147.9	32.2	3.0	2.41	NA
FL	641	Nesting	Apr-19	F	152	3.8	2.1	2.75	NA
FL	301	Nesting	Apr-19	F	156.6	21.5	5.6	2.58	NA
FL	599	Nesting	Mar-20	F	153.3	21.6	4.4	2.11	29
FL	508	Nesting	Apr-20	F	151.7	27.5	3.0	2.17	44

FL	1555	Nesting	Apr-20	F	141.7	24.2	2.8	2.87	48
FL	1556	Nesting	Apr-20	F	142.9	21.2	3.6	2.71	NA
FL	1557	Nesting	Apr-20	F	163.9	12.7	4.0	3.12	45
FL	568	Nesting	Apr-20	F	159	24.5	3.2	3.80	NA
FL	149	Nesting	Apr-20	F	160.5	18.9	2.7	3.09	30
FL	91	Nesting	Apr-20	F	163.3	41.9	3.4	3.54	35
FL	1559	Nesting	Apr-20	F	153.4	25.6	3.4	3.19	37
FL	1561	Nesting	Apr-20	F	147.4	28.7	2.9	3.13	31
FL	490	Nesting	Apr-20	F	155.7	75.6	5.7	2.16	NA
FL	1165	Nesting	Apr-20	F	158.1	28.9	2.9	3.32	32
FL	291	Nesting	Apr-20	F	154.4	10.8	1.7	2.33	64
FL	268	Nesting	Apr-20	F	147.7	36.3	4.1	2.93	NA
FL	42	Nesting	Apr-20	F	150.8	6.8	4.5	4.81	NA
FL	1563	Nesting	Apr-20	F	151.8	19.9	1.5	2.40	NA
FL	804	Nesting	Apr-20	F	139.9	14.4	4.4	2.65	33
FL	517	Nesting	Apr-20	F	146.4	10.2	3.8	3.18	27
FL	514	Nesting	Apr-20	F	146	13.3	2.9	2.83	29
FL	636	Nesting	Apr-20	F	153.9	22.3	13.0	2.98	34

Supplementary Table 1. Plasma hormone concentrations of leatherback turtles that were sampled under various circumstances. M=male, F=female, U=undetermined, CCL=curved carapace length, ALD=aldosterone, CORT=corticosterone, fT4 = free thyroxine; Time= time from initial handling until blood collection (entangled, captured), or between body pit initiation and blood collection (nesting); BLD=below limit of detection; NA=not available.
